# Enhanced fluorescent intensity of magnetic-fluorescent bifunctional PLGA microspheres based on Janus electrospraying for bioapplication

**DOI:** 10.1038/s41598-018-34856-z

**Published:** 2018-11-20

**Authors:** Kun Li, Ping Li, Zhengtai Jia, Bing Qi, Junwei Xu, Danyue Kang, Meili Liu, Yubo Fan

**Affiliations:** 1 School of Biological Science and Medical Engineering, Beihang University, Key Laboratory for Biomechanics and Mechanobiology of Ministry of Education, Beijing, 100083 China; 2 Beijing Advanced Innovation Centre for Biomedical Engineering, Beihang University, Beijing, 100083 China; 3 Department of Animal Science, College of Agriculture and Natural Resource, Michigan State University, East Lansing, MI 48824 USA; 4 Beijing Key Laboratory of Rehabilitation Technical Aids for Old-Age Disability, National Research Center for Rehabilitation Technical Aids, Beijing, 100176 China

## Abstract

Microspheres with magnetic-fluorescent functions have received attention due to fluorescent tracking and target positioning. To improve the accuracy of optical imaging and the fluorescent tracking of drug release, it is essential to enhance the fluorescent intensity of microparticles. Magnetic-fluorescent bifunctional poly lactic-co-glycolic acid (PLGA) Janus microspheres [PLGA/TbLa_3_(Bim)_12_]//[PLGA/Fe_3_O_4_] with double chambers were fabricated with the double-needle electrospraying method. The fluorescent drug TbLa_3_(Bim)_12_ with dual rare earth ions was encapsulated in one chamber, while Fe_3_O_4_ magnetic nanoparticles (Fe_3_O_4_ MNPs) were simultaneously encapsulated in another chamber. In comparison, magnetic-fluorescent PLGA composite microspheres PLGA/TbLa_3_(Bim)_12_/Fe_3_O_4_ were also prepared, which encapsulated fluorescent drugs TbLa_3_(Bim)_12_ with dual rare earth (RE) ions and Fe_3_O_4_ MNPs in one chamber. The fluorescent intensity at 542 nm of Janus microspheres was about three times higher than that of composite microspheres due to a decrease in contact between fluorescent-labeling RE drug and MNPs. The fluorescent intensities of Janus microspheres with different contents of Fe_3_O_4_ MNPs and TbLa_3_(Bim)_12_ were investigated. Furthermore, the magnetic properties, thermostability, cell toxicity and hemolytic properties of Janus microspheres were also assayed to conduct a tentative exploration of their bioapplication. The Janus microspheres provide many opportunities for application in biofields such as drug delivery.

## Introduction

With a drug delivery system (DDS), a drug can be released in a sustained or controllable manner at the target site inside the human body. However, the behavior of drug release and diffusion still poses many questions to be addressed^1,2^. Emerging functional drug delivery microspheres with unique structures or performance advantages have exhibited great potential in the field of drug delivery.

Magnetic-fluorescent functional microspheres allow targeted drug delivery by magnetic field and fluorescent tracing *in vivo*^3–5^. Maziar *et al*.^6^ synthesized fluorescent folic acid–conjugated dual thermal responsive and pH-sensitive magnetic particles as anti-cancer drug nanocarriers, realizing controlled release under simulated physiological and acidic conditions. Gu *et al*. prepared magnetic and luminescent dendrimer microspheres employing Fe_3_O_4_ magnetic nanoparticles (Fe_3_O_4_ MNPs) and CdSe/CdS quantum dot^7^.

However, in the bifunctional DDS particles, the fluorescent intensities of magnetic-fluorescent materials are greatly influenced by magnetic particles, which may cause a decrease of fluorescent intensity^8,9^. Moreover, the fluorescent marker and drugs are typically independently distributed in DDS. The separation makes it possible that the fluorescent marker may not completely reflect the drug delivery at each location or time, since the release and distribution of drugs differ from that of the fluorescent materials.

In this study, we expected to fabricate improved fluorescent tracing by preparing a fluorescent-labeled drug complex and double-chamber multifunctional microspheric DDS. It is possible to trace the behavior of drugs more accurately with a fluorescent-labeled drug. We attempted to synthesize a complex of fluorescent materials and drugs. Rare earth (RE) elements are the most common fluorescent markers that can be synthesized with drugs to obtain fluorescent-labeled drugs^10,11^. The double RE ions in the complex can increase the fluorescent intensity due to their synergetic effect of luminous RE ions and inert RE ions.

Janus structure particles with two asymmetric sides or surfaces that are different in chemical and/or physical properties provide a good solution to decrease the quenching effect of Fe_3_O_4_, and have potential applications in many fields^12–14^. Amro *et al*.^15^ fabricated novel anisotropic Janus particles with optical and magnetic properties by an oil-in-water emulsion. Budhlallc *et al*.^16^ presented the synthetic process of Janus particles containing laponite nanoclay armored with an anisotropic surface potential through the Pickering emulsion method. Shepherd *et al*.^17^ utilized microfluid devices to obtain Janus colloid-filled hydrogel granules.

Electrospraying can supply a more facile way to fabricate Janus particles with a double-needle system by using an electrostatic field force^18,19^. Ekaterina *et al*.^20^ reported the synthesis of bicompartmental microparticles containing a dual-stimuli-responsive polyethylene glycol-based polymer adopting electrospraying. Sahar *et al*.^21^ fabricated Janus^22^ microparticles composed of two distinct compartments, in which one side contained a pH responsive polymer, while the other side was composed entirely of polylactide-coglycolide (PLGA)^23^.

Via electrospraying, we prepared Janus DDS particles with magnetic and fluorescent materials in separate chambers to enhance the fluorescent intensity. Benzimidazole (Bim), applied to sterilize, relieve pain, and resist inflammation^24^, was combined with RE ions Tb^3+^ and La^3+^ to prepare the dual RE ion-labeled drug TbLa_3_(Bim)_12_. In the multifunctional Janus PLGA microspheres [PLGA/TbLa_3_(Bim)_12_]//[PLGA/Fe_3_O_4_], the Janus structure and double RE ion complexes were used to ensure fluorescent tracing. Fluorescent properties and magnetic properties of Janus microspheres with different contents and composite microspheres were studied. The thermal stability, hydrophilia and biocompatibility of Janus microspheres were tested and evaluated for possible further clinical applications.

## Methods

PLGA (M*w* = 1 × 10^5^, LA: GA = 85:15) was purchased from Jinan Daigang Biotechnology Co., LTD., China. FeCl_3_·6H_2_O (≥99.0%), FeCl_2_·4H_2_O (≥98.0%) and NaOH (≥96.0%) were obtained from Xilong Chemical Co., LTD., China. Tb_4_O_7_, La_2_O_3_ (99.99wt %), and benzimidazole (Bim, 99.99wt %) were obtained from Sinopharm Chemical Reagent Co., LTD., China. Hydrochloric acid (HCl, 36–38%), hydrogen peroxide (H_2_O_2_, 30%), chloroform (CHCl_3_, ≥99.0%) and absolute ethyl alcohol (≥99.7%) were bought from Beijing Chemical Works, China. Sprague Dawley (SD) rats were obtained from the Peking University Healthcare Science Center. DMEM culture medium was purchased from Thermo Fisher Scientific, USA. Fetal bovine serum (FBS) was obtained from Tianjin Kang Yuan Biological Technology Co., LTD., China. Polylysine was purchased from Sigma-Aldrich Co. LLC., USA. A Cell Counting Kit-8 (CCK8) was acquired from Beyotime Institute of Biotechnology Co., LTD., China. Trypsin, cytosine arabinoside, penicillin, streptomycin sulphate and a bicinchoninic acid (BCA) Protein Assay Kit were obtained from Beijing Solarbio Life Science Co., LTD., China. Phosphate buffer solution (PBS) was prepared in the laboratory. The Fe_3_O_4_ MNPs were prepared according to a previous study^25^.

RE fluorescent-labeled drugs Tb(Bim)_3_ and TbLa_3_(Bim)_12_ were prepared according to our previous study^26^. TbCl_3_ was synthesized by dissolving Tb_4_O_7_ in a concentrated HCl solution and H_2_O_2_ solution under vigorous stirring at 60 °C until the solution became thoroughly clear. LaCl_3_ was synthesized with a similar method.

Specific amounts of TbCl_3_ and Bim were separately dissolved in absolute ethyl alcohol. When synthesizing Tb(Bim)_3_, the Bim ethyl alcohol solution was added slowly into the TbCl_3_ ethyl alcohol solution at a molar ratio of 3:1 under magnetic stirring at 60 °C for 4 h. Tb(Bim)_3_ was brought to 6.5–7 pH by NaOH. The fluorescent-labeled drug TbLa_3_(Bim)_12_ with dual RE ions Tb^3+^ and La^3+^ was prepared with a similar method at the molar ratio of TbCl_3_, LaCl_3_ and Bim of 1: 3: 12. The yield of complex TbLa_3_(Bim)_12_ with RE ions was approximately 20–30%.

Magnetic-fluorescent bifunctional [PLGA/TbLa_3_(Bim)_12_]//[PLGA/Fe_3_O_4_] Janus microspheres and PLGA/TbLa_3_(Bim)_12_/Fe_3_O_4_ composite microspheres were fabricated via electrospraying. TbLa_3_(Bim)_12_ and Fe_3_O_4_ MNPs were dispersed in 2.5 mL CHCl_3_ via ultrasound. Then, 0.1 g PLGA was added to the above dispersions under magnetic stirring for 12 h to obtain spraying dispersion A and B. The amounts of TbLa_3_(Bim)_12_ and Fe_3_O_4_ MNPs for different samples are illustrated in Table 1. The spraying dispersion of composite microspheres was prepared by dispersing 0.1 g TbLa_3_(Bim)_12_ and 0.1 g Fe_3_O_4_ MNPs in 5 mL CHCl_3_, and 0.2 g PLGA was added to the above dispersion under 12 h magnetic stirring.

**Table 1:** Compositions of sprayed dispersions of Janus microspheres [PLGA/TbLa_3_(Bim)_12_]//[PLGA/Fe_3_O_4_].

Sample	Method	PLGA/g		CHCl_3_/mL	TbLa_3_(Bim)_12_/g	Fe_3_O_4_ MNPs/g
S1	Double needles	A	0.1	2.5	0.25	
		B	0.1	2.5		0.10
S2	Double needles	A	0.1	2.5	0.05	
		B	0.1	2.5		0.10
S3	Double needles	A	0.1	2.5	0.10	
		B	0.1	2.5		0.10
S4	Double needles	A	0.1	2.5	0.10	
		B	0.1	2.5		0.05
S5	Double needles	A	0.1	2.5	0.10	
		B	0.1	2.5		0.02

**Figure 1:**
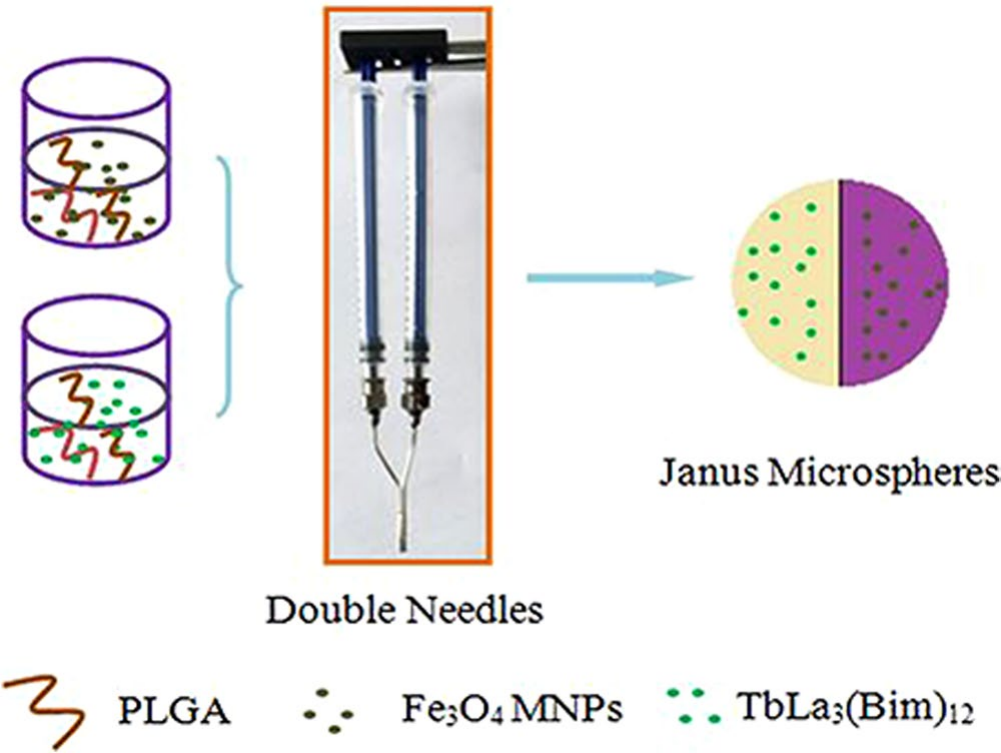
Schematic of the preparation of Janus microspheres [PLGA/TbLa_3_(Bim)_12_]//[PLGA/Fe_3_O_4_].

As shown in Fig. 1, [PLGA/TbLa_3_(Bim)_12_]//[PLGA/Fe_3_O_4_] Janus microspheres were prepared by injecting dispersion A and B in the double needles of a parallel sprayer. Aluminum foil was employed as a collector and shelved 30 cm away from the tip of the stainless steel needles to collect microspheres. A positive direct current voltage of 7 kV was applied between the sprayer and the collector. Moreover, flow rates of dispersion A and B remained at 0.4 mL/h during electrospraying. All procedures were conducted at room temperature. Furthermore, composite microspheres were prepared using a single needle electrospraying setup, and the parameters of electrospraying were identical to those of Janus microspheres.

The structures of Bim, Tb(Bim)_3_, and TbLa_3_(Bim)_12_ were investigated via an FTIR spectrometer (FTIR-650, Tianjin Gang Dong Technology Development Co., LTD, China) and UV/VIS/NIR spectrophotometer (UV-3600, SHIMADZU, Japan). The constitution of Fe_3_O_4_ MNPs, Bim, Tb(Bim)_3_, TbLa_3_(Bim)_12_, Janus microspheres (S3), and composite microspheres was measured through an X-ray diffractometer (XRD, D/MAX-2500, RIGAKU, Japan) at 40 kV and 200 mA.

The morphologies of Janus microspheres (S3) were observed via a scanning electron microscope (SEM) (Quanta^TM^, FEG 250, SEM, FEI, USA) and transmission electron microscope (JEM 1200EX, TEM, NEC Electronics Corporation, Japan). The sizes of the Janus microspheres and composite microspheres were calculated using 100 samples from SEM images.

The fluorescent properties of all samples were measured via a fluorescence spectrophotometer (FS5, Edinburgh, UK). The Janus microspheres (S5) were dispersed in absolute ethyl alcohol in a 12-well culture plate, and the fluorescent image was obtained using a fluorescence microscope (FM, IX73, Olympus, Japan).

The magnetic properties of Janus microspheres (S3-S5) and composite microspheres were studied by using a vibrating sample magnetometer (VSM, 7307, Lake Shore, USA). The magnetic responses of 0.02 g Fe_3_O_4_ MNPs in PBS or 4% PLGA solution and 0.02 g Janus microspheres in PBS were observed using a magnet.

The thermodynamic properties of Janus microspheres (S3) and composite microspheres were measured using a thermal gravimetric analyzer (TGA, METTLER TGA/DSC1 SF/1382, HITACHI, Japan) at a heating rate of 10 °C/min.

The Zeta potentials of Janus microspheres (S3) dispersed in water (1 mg/mL) at 0, 1, 4 and 7 d were detected via a laser particle analyzer (Zetasizer Nano ZS, Malvern, UK).

The contact angles of the PLGA membrane, PLGA microsphere film, composite microsphere film, and Janus microsphere film were surveyed via a contact angle measurement instrument (JC2000FM, Shanghai Zhong Chen Digital Technology Device Co., LTD., China). The PLGA membrane was obtained by dropping 4% (m/v) PLGA CHCl_3_ solution onto aluminum foil (1 × 1 cm) until solidification. PLGA microsphere, composite microsphere, and Janus microsphere (S3) films were electrosprayed at a concentration of 4% (0.4 mL/h, 30 cm, 7 kV) for 2.5 h. All samples were placed under the microliter syringes (100 μL), and deionized water droplets were randomly deposited onto the films. The contact angles were measured through a five-point fitting method and a built-in calculation software according to the images captured by the camera. Every sample was measured at least in triplicate.

The cytocompatibility of Janus microspheres (S3) was assayed with rat cerebral cortex nerve cells. Newborn Sprague Dawley (SD) rats were decapitated; the cerebral cortex was removed, separated, and collected in DMEM culture medium containing 0.1 g penicillin and 0.1 g streptomycin sulphate. The tissue was cut into slivers, and approximately 3 mL trypsin was added for digestion. Next, the above mixture was transferred to an incubator at 37 °C and 5% CO_2_ atmosphere for approximately 30 min, and 10% FBS was added to terminate the digestion. DMEM was injected into the mixture, and the mixture was filtered; then, the filter liquor was centrifuged at 1000 rpm for 5 min, and the sediment consisted of nerve cells. The precipitated cells were resuspended and incubated in 96-well plates that were coated with polylysine. Cell viability was measured by using a CCK-8 test. The rat cerebral cortex nerve cells in 96-well plates were cultured with medium containing Janus microspheres at concentrations from 0.01, 0.1, 1 to 100 μg/mL. After 24 h incubation, the medium was removed, and the CCK-8 solution combined with medium was added at a volume ratio of 1:9. Then, the 96-well plate was incubated for 30 min and detected via a microplate reader (VARIOSKAN FLASH, Thermo Scientific, USA).

A hemolytic assay with Janus microspheres (S3) was performed according to a previous report^27^. EDTA-stabilized rabbit blood samples were obtained from Peking University Health Science Center (Beijing, China). The blood was centrifuged at 2000 rpm for 5 min, and the remaining red blood cell (RBC) pellet was washed with PBS until the supernatant became transparent. Janus microspheres were dispersed in sodium chloride injection solution at different concentrations from 0.01, 0.1, 1, 10 to 100 μg/mL. Then, 0.8 mL of the Janus microsphere dispersion was mixed with 0.2 mL of 2% RBC PBS suspension. Moreover, 0.8 mL of a water and sodium chloride injection was added to 0.2 mL of the RBC suspension as positive and negative control samples. All samples were incubated at room temperature for 2 h and then centrifuged at 2000 rpm. Then, 100 mL of supernatant was extracted for absorbance detection at 570 nm via a microplate reader (VARIOSKAN FLASH, Thermo Scientific, USA). Hemolysis was defined as follows:

$${\rm{Hemolysis}}=({{\rm{absorbance}}}_{{\rm{eg}}}-{{\rm{absorbance}}}_{{\rm{ng}}})/({{\rm{absorbance}}}_{{\rm{pg}}}-{{\rm{absorbance}}}_{{\rm{ng}}})$$

where eg represents the experimental group, ng represents the negative group, and pg represents the positive group.

A BCA assay kit was used to evaluate the protein binding of Janus microspheres. Briefly, the BCA working reagent was prepared by mixing the BCA stock solution with 4% CuSO_4_∙5H_2_O at a volume ratio of 50:1 prior to beginning the assay. Then, 1 mL bovine serum albumin protein standard (0.5 mg/mL) and Janus microspheres with different contents (0.1, 1, 10 and 100 μg/mL) were mixed and kept in ultrasonic dispersion for 2 h. The mixture was centrifuged and washed twice with PBS to obtain the sample absorbing protein. Then, 1 mL PBS was added to the above samples absorbing protein with adequate blending to obtain the protein samples. The 20 μL protein samples were mixed with 200 μL BCA working reagent and incubated at 37 °C for 30 min before being read at 562 nm.

Statistical analysis of data was performed via one-way analysis of variance (ANOVA), assuming a confidence level of 95% (p < 0.05) for statistical significance through SPSS 19.0.

### Ethical approval

All methods and experimental protocols for the care and use of animals were performed in accordance with the relevant guidelines and regulations and approved by the Animal Welfare and Ethics Branch, Biomedical Ethics Committee of Peking University.

## Results and Discussion

### IR analysis

**Figure 2:**
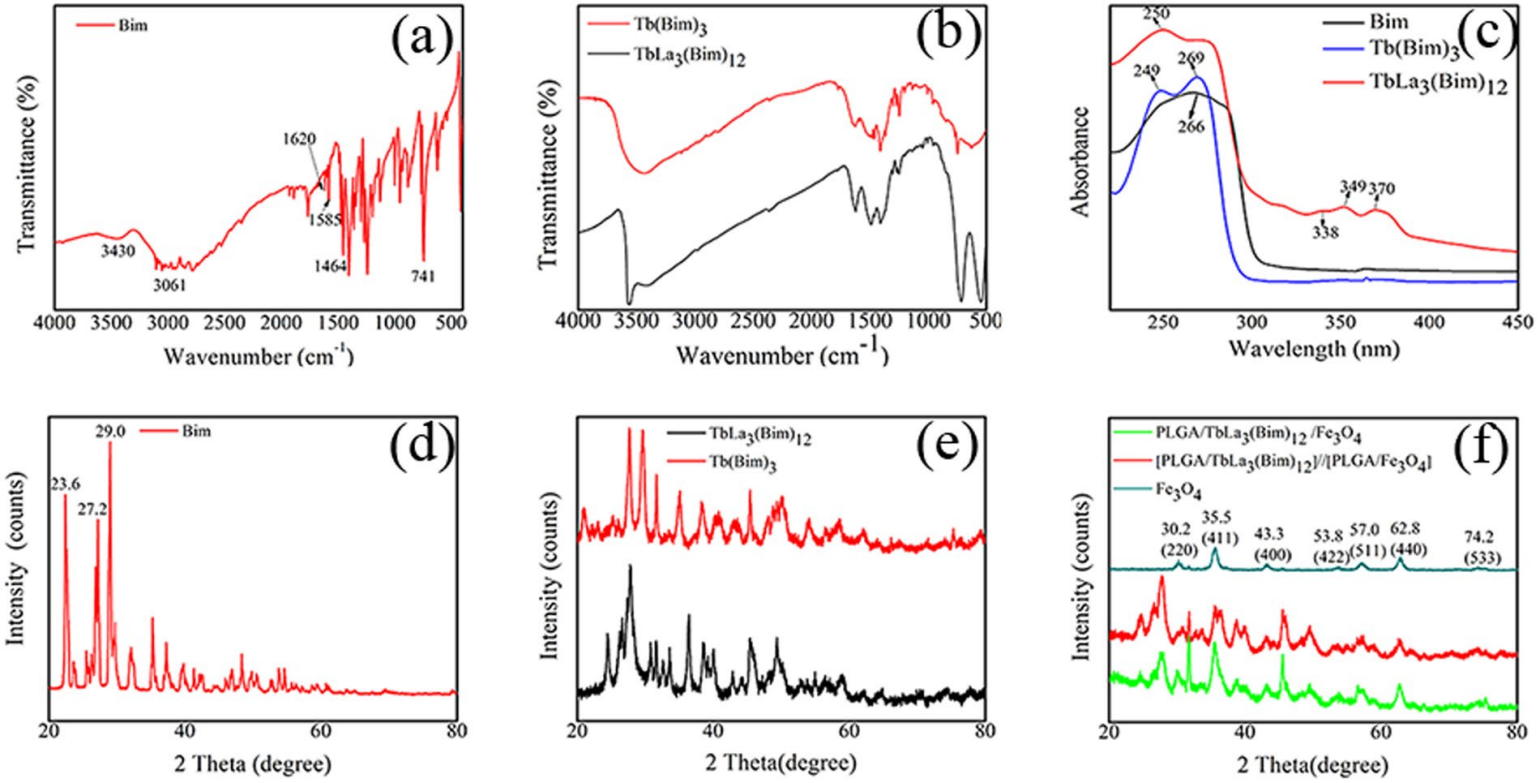
FTIR spectra of (**a**) Bim and (**b**) Tb(Bim)_3_ and TbLa_3_(Bim)_12_. UV spectra of (**c**) Bim, Tb(Bim)_3_, and TbLa_3_(Bim)_12_. XRD patterns of (**d**) Bim, (**e**) TbLa_3_(Bim)_12_ and Tb(Bim)_3,_ (**f**) Fe_3_O_4_ MNPs, Janus microspheres (S3) and composite microspheres.

In Fig. 2(a), the peaks located at 3061, 1620, and 1585 cm^−1^ were respectively derived from C–H unsaturated stretching vibration in the aromatic ring, C=C stretching vibration in the imidazole ring, and C=N skeletal vibration. Peaks at 1464 and 741 cm^−1^ represented the C-N stretching vibration in the imidazole ring and C-H out-of-plane bending vibration of Bim. Figure 2(b) shows that the peaks located at 1585 and 1464 cm^−1^ shifted to 1504 and 1405 cm^−1^, respectively. The above results could be caused by the interaction between nitrogen atoms in the imidazole ring and RE ions Tb^3+^, La^3+^ ^28^. The new peak situated at 543 cm^−1^ was attributed to Tb-N and La-N stretching vibration^29^. The intensity of the C-H out-of-plane bending vibration peak at 733 cm^−1^ of TbLa_3_(Bim)_12_ was stronger than that of Tb(Bim)_3_, which may be related to the addition of La^3+^. The characteristic broad peak in 3430 cm^−1^ of the O-H vibration indicated the existence of crystal water in the coordination compounds TbLa_3_(Bim)_12_ and Tb(Bim)_3_.

### UV analysis

In Fig. 2(c), the characteristic absorption peak of ligand Bim at 266 nm was from absorption of the π-π* transition of the aromatic ring. The peak of Bim located at 266 nm shifted to 250 and 249 nm in TbLa_3_(Bim)_12_ and Tb(Bim)_3_, respectively. This shift could be due to the increased rigidities of TbLa_3_(Bim)_12_ and Tb(Bim)_3_ caused by the coordination reaction between Tb^3+^, La^3+^, and Bim^30^. The shifts of the absorption peaks indicated an interaction between Tb^3+^, La^3+^ and Bim. In TbLa_3_(Bim)_12_, peaks at 338, 349, and 370 nm corresponded to the 4f-4f transition of Tb^3+^ in which electrons were excited from the ^7^F_6_ ground state to the higher 4 f energy level.

### XRD analysis

The XRD patterns of Tb(Bim)_3_, TbLa_3_(Bim)_12_, Fe_3_O_4_ MNPs, Janus microspheres, and composite microspheres are shown in Fig. 2(d–f). Compared to the patterns of Tb(Bim)_3_ and TbLa_3_(Bim)_12_, the peak of Bim located at 27.2° shifted to 27.6° in TbLa_3_(Bim)_12_ and 27.8° in Tb(Bim)_3_, and the peak of Bim located at 23.6° disappeared. The peak situated at 29.0° did not exhibit an obvious shift in Tb(Bim)_3_ but shifted to 30.6° in TbLa_3_(Bim)_12_. These phenomena indicated that the addition of RE ions Tb^3+^ and La^3+^ had an effect on crystallization properties and structures. In the XRD patterns of Fe_3_O_4_ MNPs, the peaks seated at 30.5°, 35.5°, 43.1°, 53.5°, 57.0°, 62.8°, and 74.2° corresponded to (220), (311), (400), (422), (511), (440), and (533), respectively. The reflection planes of the face-centered cubic Fe_3_O_4_ crystal of Fe_3_O_4_ MNPs showed that Fe_3_O_4_ MNPs contained no impurities and exhibited high degrees of crystallinity. Figure 2(f) also shows that the XRD patterns of both Janus microspheres and composite microspheres demonstrated the characteristic peaks of Fe_3_O_4_ MNPs and TbLa_3_(Bim)_12_. It was concluded that Fe_3_O_4_ MNPs and TbLa_3_(Bim)_12_ were both wrapped in the Janus microspheres and composite microspheres.

### Morphologies

**Figure 3:**
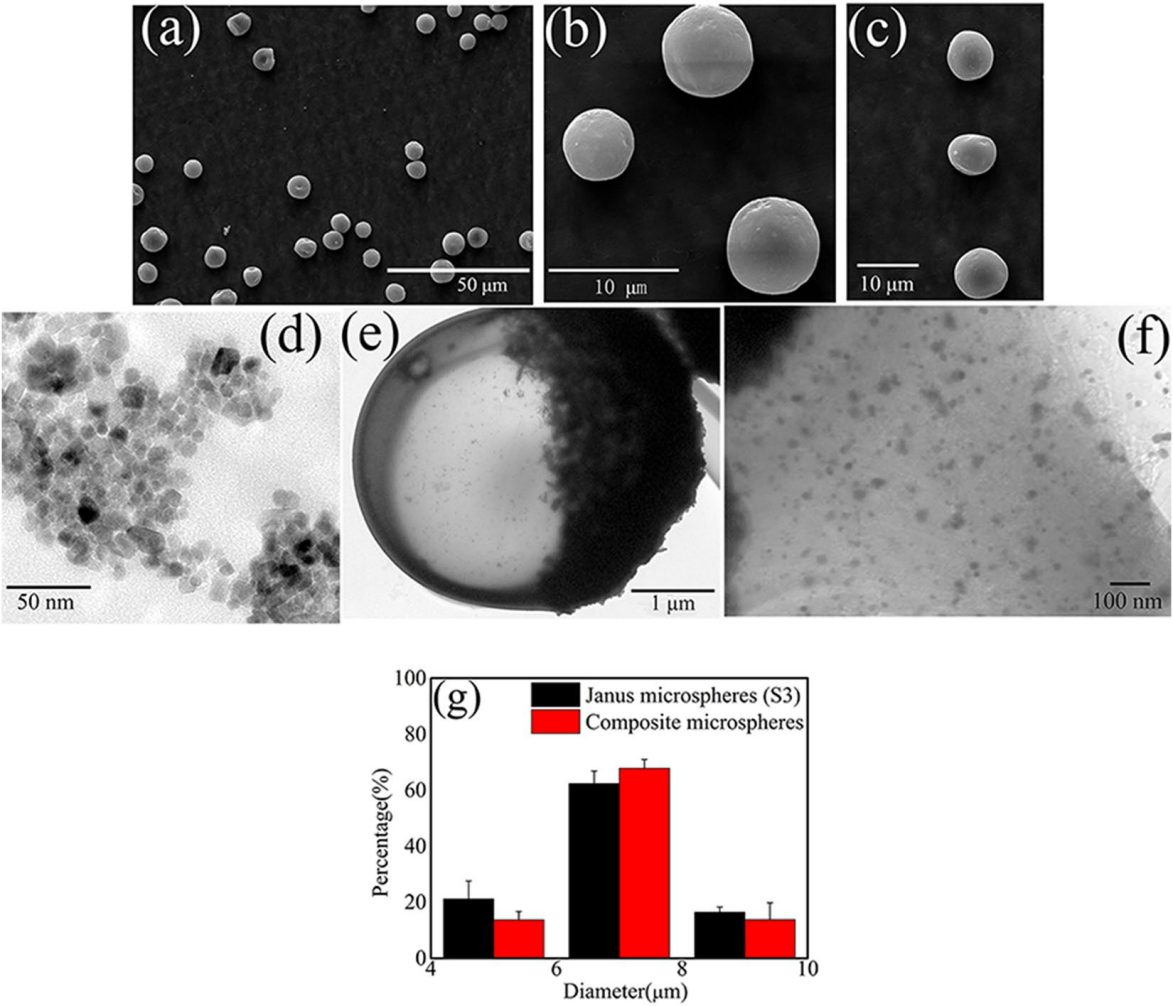
SEM images of (**a,b**) Janus microspheres (S3) containing 0.10 g TbLa_3_(Bim)_12_ and 0.10 g Fe_3_O_4_ MNPs, and (**c)** composite microspheres. TEM images of (**d**) Fe_3_O_4_ MNPs, (**e**) Janus microspheres (S3), and (**f**) enlargement of the left chamber of (TbLa_3_(Bim)_12_) particles in (**e**). (**g**) Particle size distribution of Janus microspheres (S3) and composite microspheres.

Janus microspheres and composite microspheres containing 0.1 g TbLa_3_(Bim)_12_ and Fe_3_O_4_ MNPs were observed under SEM, exhibiting good dispersibilities and excellent spherical shapes, as shown in Fig. 3(a–c). The diameter of Fe_3_O_4_ MNPs modified by HCl remained below 10 nm, and Fe_3_O_4_ MNPs showed good dispersibility as seen in Fig. 3(d). Due to the large amount of Fe-OH on the surface of Fe_3_O_4_ MNPs and more H^+^ absorbed to the surface that produced stronger electrostatic repulsion, the dispersibility of Fe_3_O_4_ MNPs was enhanced after modification by HCl^25^. The structure of a single Janus microsphere was studied under TEM, as shown in Fig. 3(e). The Janus microsphere possessed two obvious chambers with different colors, which indicated that Fe_3_O_4_ MNPs and TbLa_3_(Bim)_12_ particles distributed into two different compartments. This good Janus structure is beneficial for the fluorescent performance of DDS microspheres. The left part of the particle in Fig. 3(e) was enlarged and is shown in Fig. 3(f), and the TbLa_3_(Bim)_12_ particles were found to be evenly dispersed in PLGA particles. The particle size distribution in Fig. 3(g) showed no significant difference between the Janus and composite microspheres, and the sizes of Janus and composite microspheres were mostly approximately 6–8 μm.

### Fluorescent property

**Figure 4:**
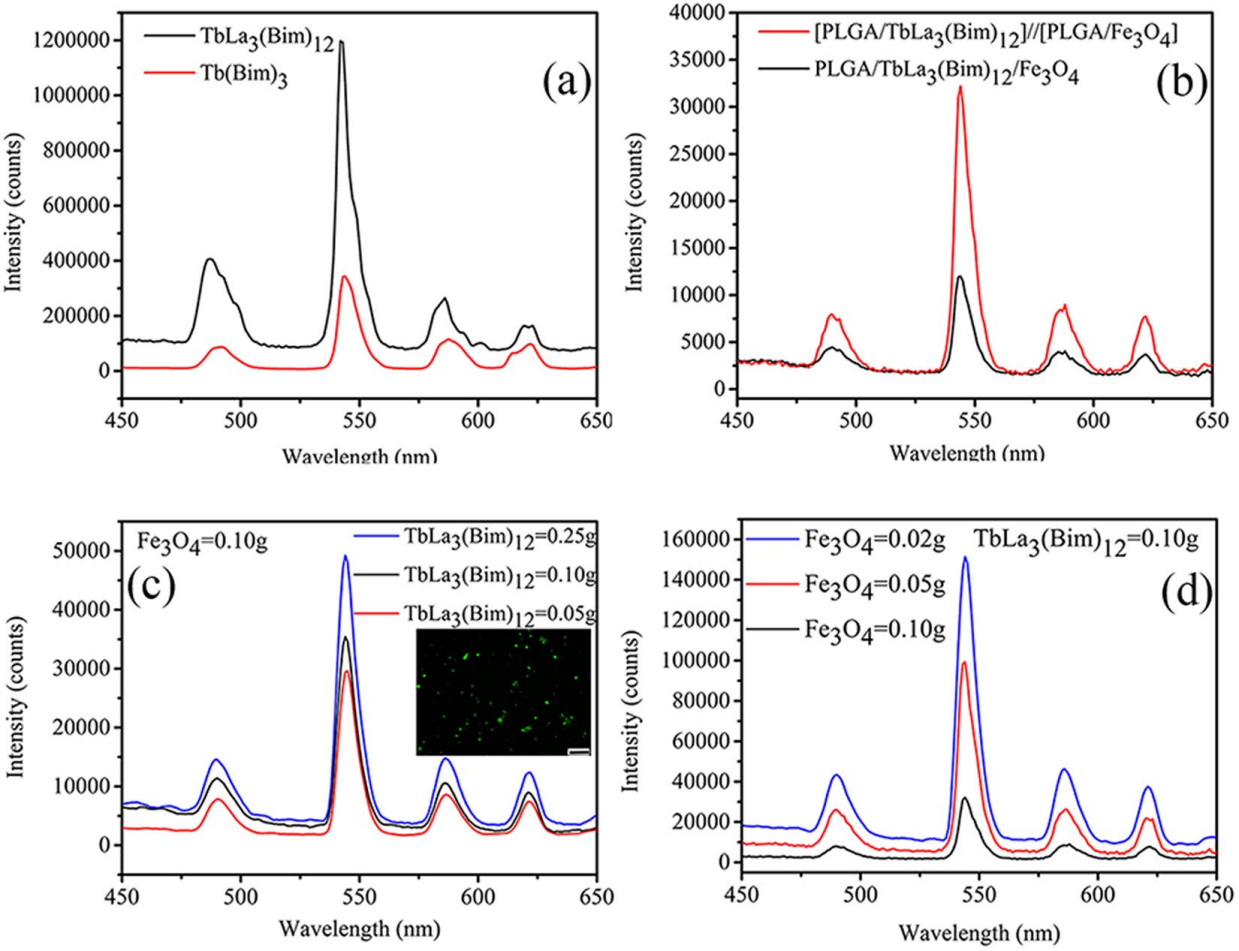
Emission spectra of (**a**) Tb(Bim)_3_ and TbLa_3_(Bim)_12_, (**b**) Janus microspheres (S3) and composite microspheres, (**c**) Janus microspheres with various contents of TbLa_3_(Bim)_12_ at a 0.1 g Fe_3_O_4_ MNP mass (the inserted fluorescent image was derived from Janus microspheres S5, the scale bar is 50 μm) and (**d**) Janus microspheres with different masses of Fe_3_O_4_ MNPs at 0.1 g TbLa_3_(Bim)_12_.

The emission spectra of Tb(Bim)_3_ and TbLa_3_(Bim)_12_ were described when the excited wavelength was fixed at 270 nm in Fig. 4(a). Emission spectra of both Tb(Bim)_3_ and TbLa_3_(Bim)_12_ exhibited characteristic peaks at 487, 542, 586, and 621 nm, corresponding to Tb^3+^ energy level transitions of ^5^D_4_–^7^F_6_, ^5^D_4_-^7^F_5_, ^5^D_4_-^7^F_4_, and ^5^D_4_-^7^F_3_, respectively. The fluorescent intensity of TbLa_3_(Bim)_12_ was much higher than that of Tb(Bim)_3_. The second RE ion La^3+^, whose outermost electron configuration was ^5^S_2_^5^P_6_, was brought into the RE complex. The excited level of La^3+^ possessed a fairly stable structure due to the high excited level of La^3+^. The energy absorbed by Bim was not consumed through radiative transition of La^3+^, but it would be transferred to Tb^3+^ through the bridge connection between Bim and Tb^3+^/La^3+^. Thus, the total energy absorbed by Tb^3+^ was enhanced and the fluorescent intensity of TbLa_3_(Bim)_12_ was stronger than that of Tb(Bim)_3_.

The spectra presented in Fig. 4(b) showing the fluorescent intensities of Janus and composite microspheres were investigated. The fluorescent intensity of Janus microspheres was far stronger than that of composite microspheres. Composite microspheres were manufactured via single needle, and TbLa_3_(Bim)_12_ and Fe_3_O_4_ MNPs were distributed evenly in microspheres. Janus microspheres were prepared using parallel double needles, and TbLa_3_(Bim)_12_ and Fe_3_O_4_ MNPs were distributed independently in two chambers of the spheres. Therefore, the contact between TbLa_3_(Bim)_12_ and Fe_3_O_4_ MNPs decreased, and the fluorescent signals assimilated by magnetic particles were lessened. Consequently, the fluorescent intensity of the Janus microspheres was greatly enhanced compared to the composite microspheres. At 542 nm, the fluorescent intensity of Janus microspheres was about three times higher than that of composite microspheres.

Figure 4(c) shows the fluorescent spectra of Janus microspheres containing different contents of TbLa_3_(Bim)_12_. With 0.10 g Fe_3_O_4_ MNPs, the fluorescent intensities of Janus microspheres increased gradually with the increasing contents of TbLa_3_(Bim)_12_ from 0.05 to 0.25 g. The appropriate content of TbLa_3_(Bim)_12_ was fairly important to prepare DDS microspheres with good fluorescent performance. Hence, it was necessary to optimize the content of RE complexes and achieve optimal fluorescent performance. The image inserted in Fig. 4(c) shows the fluorescent image of Janus microspheres. The fluorescent image launched a distinct green fluorescence, which was the characteristic color fluorescence of Tb^3+^. The bright fluorescence of Janus microspheres played a significant role in field labeling.

The emission spectra of Janus microspheres with different contents of Fe_3_O_4_ MNPs are shown in Fig. 4(d). The fluorescent intensities of Janus microspheres with different contents of Fe_3_O_4_ MNPs from 0.02 to 0.10 g decreased gradually when the content of TbLa_3_(Bim)_12_ was fixed at 0.10 g. Due to the capability of black Fe_3_O_4_ MNPs to assimilate the fluorescence, the fluorescent intensity of Janus microspheres was enhanced along with the decreasing content of Fe_3_O_4_ MNPs.

Due to the characteristic Janus structure, Janus microspheres presented good fluorescent properties compared to the composite microspheres. The contents of TbLa_3_(Bim)_12_ and Fe_3_O_4_ MNPs could exerted a strong impact on the fluorescent intensity of Janus microspheres.

### Magnetic analysis

**Figure 5:**
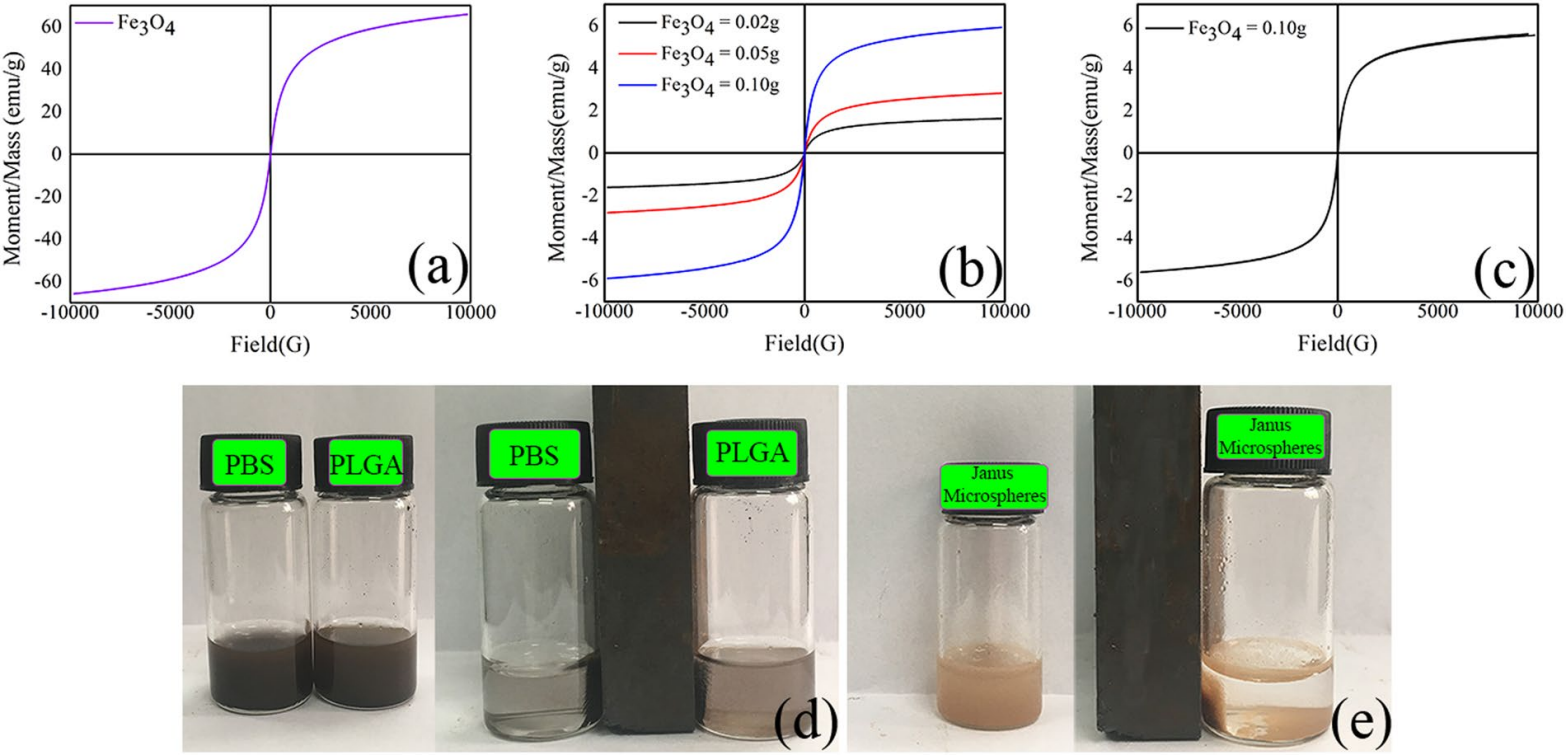
Hysteresis loop of (**a**) Fe_3_O_4_ MNPs, (**b**) Janus microspheres (S3-S5), and (**c**) composite microspheres. Magnetic responses of (**d**) 0.02 g Fe_3_O_4_ MNPs in PBS or 4% PLGA solution, and (**e**) 0.02 g Janus microspheres (S3) in PBS solution.

The hysteresis loops of Fe_3_O_4_ MNPs, composite microspheres and Janus microspheres containing different contents of Fe_3_O_4_ MNPs are presented in Fig. 5. Fe_3_O_4_ MNPs exhibited superparamagnetism with no magnetic remnants and zero coercive force. The saturation magnetizations of all microspheres were below that of Fe_3_O_4_ MNPs. This was possibly caused by introduction of PLGA and TbLa_3_(Bim)_12_ and decreasing contents of Fe_3_O_4_ MNPs per unit mass. The saturation magnetization of Janus microspheres showed an increasing trend along with the contents of Fe_3_O_4_ MNPs from 0.02 to 0.10 g, and the saturation magnetization reached 5.9088 emu/g, which was similar to that of the composite microspheres (5.6098 emu/g). This finding indicated that the Janus structure showed no difference in the magnetic properties of two types of microspheres.

In Fig. 5(d,e), magnetic responses of 0.02 g Fe_3_O_4_ MNPs in PBS or 4% PLGA solution, and 0.02 g Janus microspheres in PBS were evaluated. From Fig. 5(d), Fe_3_O_4_ MNPs in PBS and 4% PLGA solution were quickly gathered in the area where the magnet was placed after 10 s. However, Fe_3_O_4_ MNPs in 4% PLGA solution was collected more slowly than that in PBS, which was a result of the higher viscosity of the 4% PLGA solution than PBS. In Fig. 5(e), the Janus microspheres were also rapidly collected at the area where the magnet was placed after 10 s, which indictaed the Janus microspheres possessed good magnetic response.

### Thermodynamic analysis

**Figure 6:**
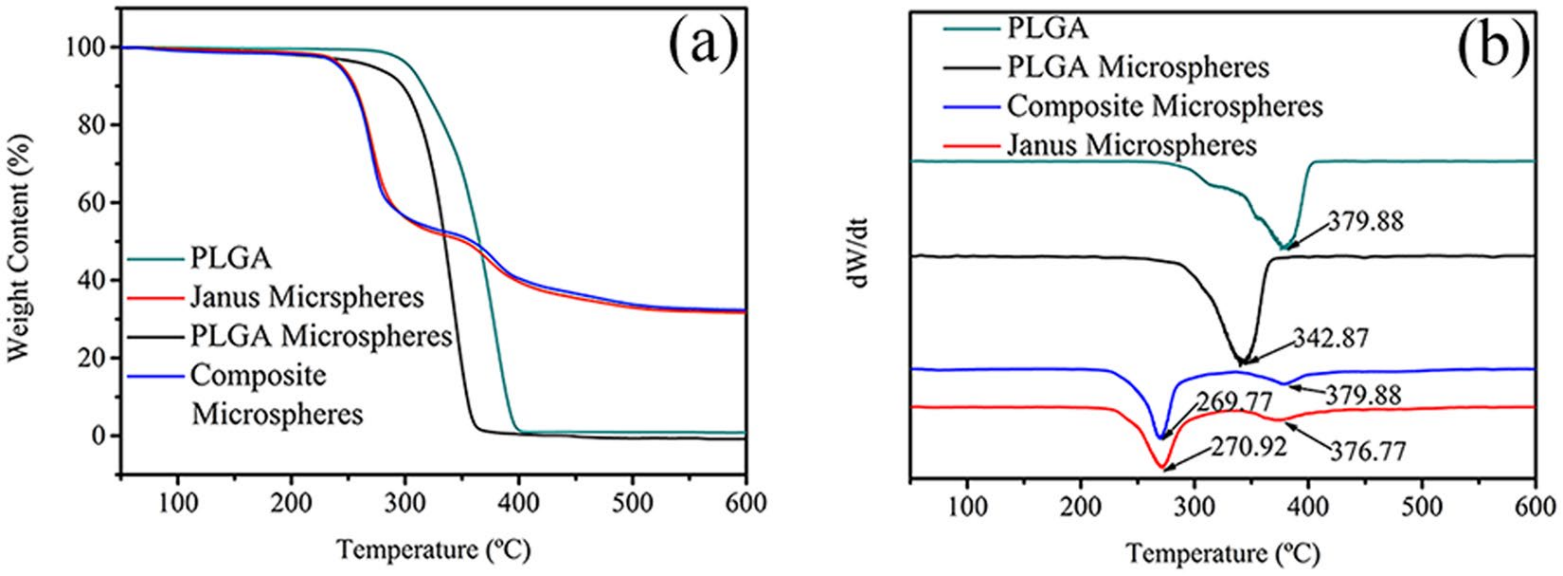
TG (**a**) and DTG (**b**) curves of pure PLGA, PLGA microspheres, composite microspheres, and Janus microspheres (S3).

TG and DTG curves are depicted in Fig. 6. In Fig. 6(a), the weights of pure PLGA and PLGA microspheres underwent a loss stage and were close to zero after decomposition of PLGA. The weights of composite microspheres and Janus microspheres both experienced two loss stages, which represented the decomposition of PLGA and Bim. The remaining weight (approximately 32%) was the weight of Fe_3_O_4_ MNPs and Tb^3+^, La^3+^. Maximum weight loss rates of pure PLGA solid, PLGA microspheres, composite microspheres, and Janus microspheres are shown in Fig. 6(b), which occurred at 379.88, 342.87, 269.77, and 270.92 °C, respectively. Compared to pure PLGA solid, the decomposition temperature of PLGA microspheres declined. The reason could be that PLGA microspheres were prepared in a high-voltage electrostatic field, which might destroy the PLGA chain structure and decrease the thermal stability of PLGA microspheres. Composite microspheres and Janus microspheres showed a further decline in decomposition temperature, which was possibly caused by the addition of Fe_3_O_4_ MNPs and TbLa_3_(Bim)_12_ fluorescent-labeling drug. Furthermore, the DTG curves of composite microspheres and Janus microspheres exhibited a peak at 379.88 and 376.77 °C, respectively, due to the decomposition of Bim.

The TG and DTG curves of composite microspheres and Janus microspheres were quite similar, indicating that the difference of structure did not impact the thermal performance of both types of microspheres. Both showed good thermal stability, which was important to their application in DDS.

### Zeta potential evaluation

The zeta potentials of Janus microspheres (S3) dispersing in water at 0, 1, 4 and 7 d are shown in Table 2. Zeta potential could reflect the stability of the dispersion system according to the interaction among particles. We found that the Zeta potential of the engineered particles did not obviously change from 0 to 7 d, which proved the stability of the Janus microspheres in dispersion.

**Table 2:** Zeta potentials of Janus microspheres (S3) dispersed in water after 0, 1, 4 and 7 d.

Time	Zeta potential (mV)
0 d	−14.0 ± 4.40
1 d	−13.1 ± 4.22
4 d	−13.3 ± 4.54
7 d	−10.6 ± 5.27

### Contact angle analysis

**Figure 7:**
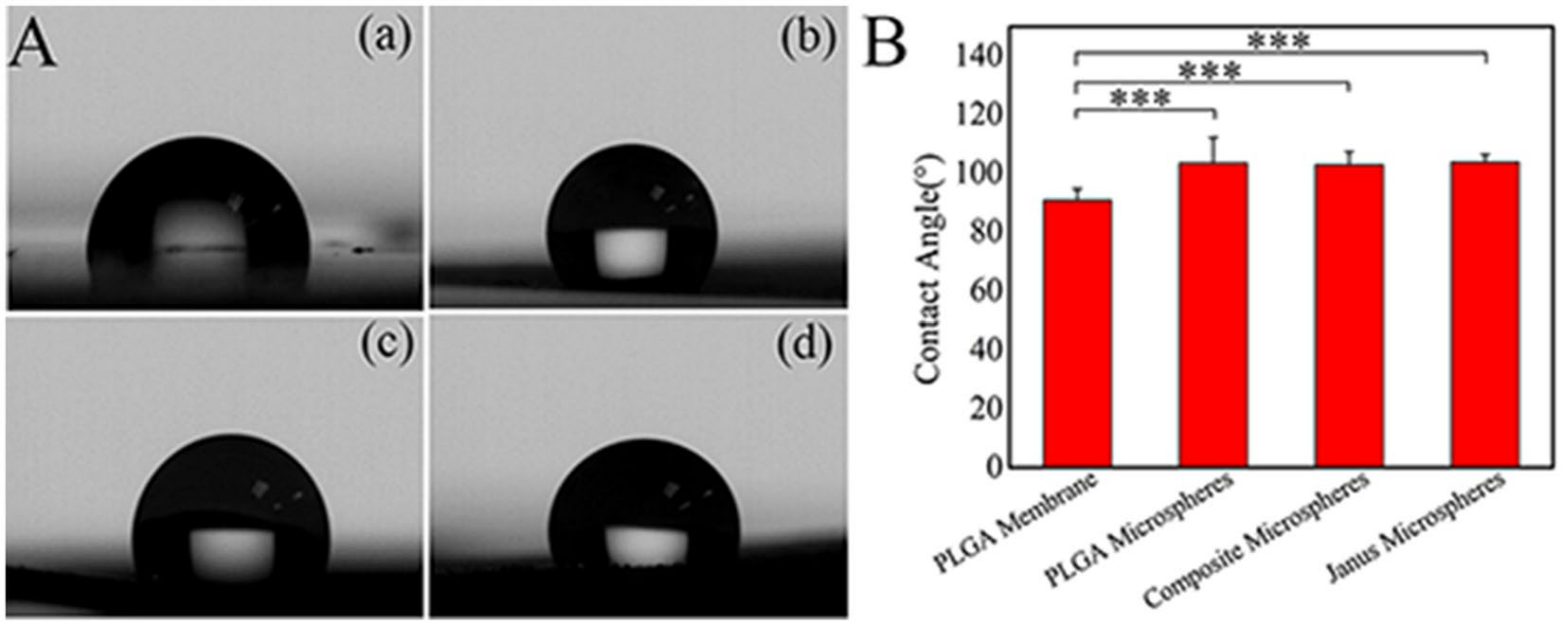
Contact angle measurement results. A: Contact angle images of the (**a**) PLGA membrane, (**b**) PLGA microsphere film, (**c**) composite microsphere film and (**d**) Janus microsphere (S3) film. B: Contact angles of the PLGA membrane, PLGA microsphere film, composite microsphere film, and Janus microsphere film (_***_ represents p < 0.01).

As shown in Fig. 7, the contact angle of the PLGA film was 93.5°. The contact angles of the PLGA microsphere films, composite microsphere films, and Janus microsphere films were all approximately 105.6°, and did not exhibit much difference. The hydrophobicity of microsphere films was higher than that of PLGA films. This may be caused by the different roughness between cast film and microsphere films^31,32^. Furthermore, the degradation of drug delivery microspheres was mainly achieved via hydrolysis of PLGA. Improved hydrophobic property lessened the PLGA degradative capability and prolonged the drug delivery time to some extent.

### Cell toxicity evaluation

**Figure 8:**
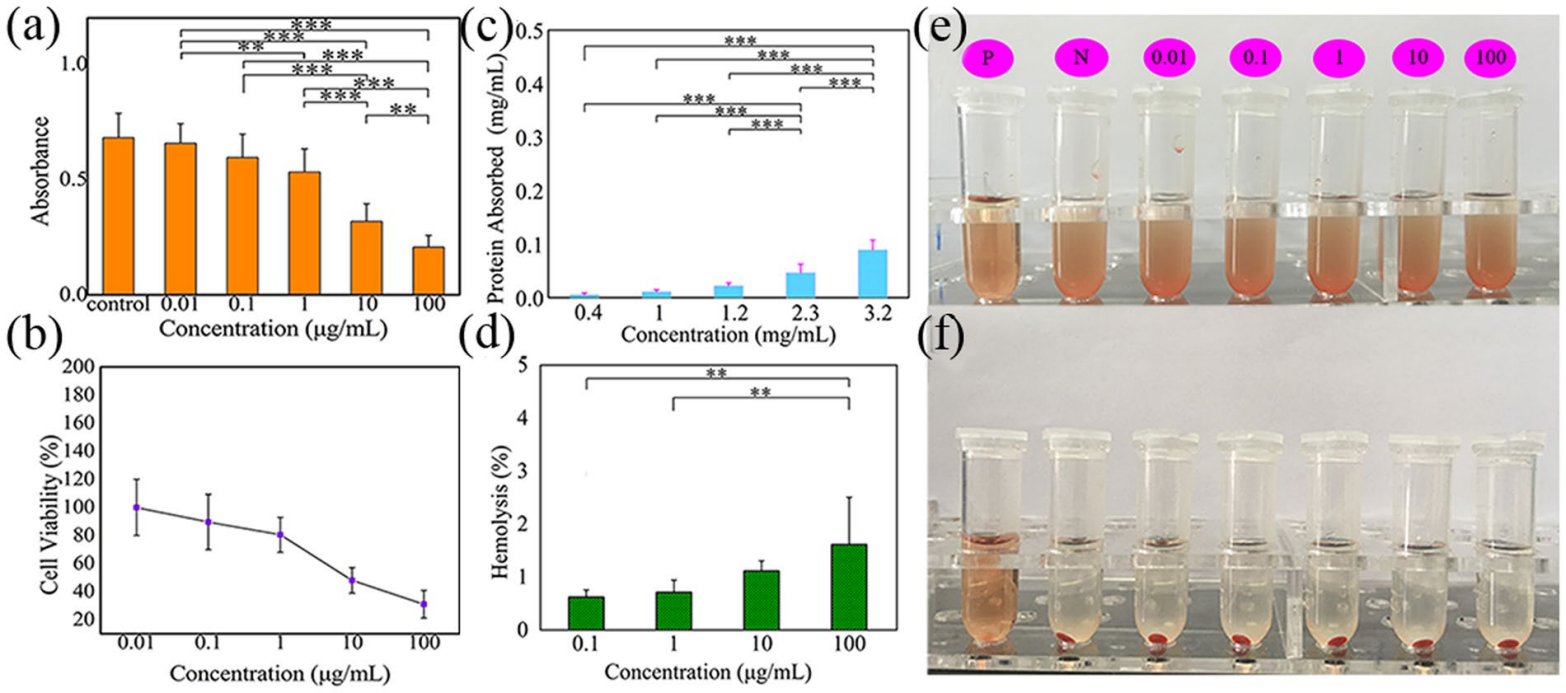
Cell toxicity, protein adsorption evaluation results, and hemolytic results. (**a**) The absorbance of cells cultured with Janus microspheres (S3) at different concentrations for 2 d. (**b**) Cell viability of cells cultured with Janus microspheres. (**c**) Protein adsorption ratios of different concentrations of Janus microspheres (S3). (**d**) Hemolysis ratios of different concentrations of Janus microspheres (S3). (**e**) Image of red blood cells mixed with Janus microspheres (S3). (**f**) Image of red blood cells with Janus microspheres (S3) after 2 h centrifugation (P represents the positive control, N represents the negative control, ^***^represents p < 0.01, ^**^represents p < 0.05, bars correspond to the means ± standard deviation for n = 5 measurements).

Cell toxicity evaluation is one of the basic preconditions before biomaterials can be applied in clinical tests. The cell viability after cells were cocultured with Janus microspheres is shown in Fig. 8(a,b). Compared to the control group, all cell viabilities declined when cells were incubated in media with different contents of Janus microspheres. However, when the concentrations of Janus microspheres ranged from 0.01 to 1 μg/mL, the cell viability exceeded 80%. Moreover, compared to the concentration of 0.01 μg/mL, the cell viability exhibited a significant decrease when the concentration was 1 μg/mL; however, there was no obvious difference between the 0.01 μg/mL and 0.1 μg/mL group. This finding indicated that the dispersion with concentrations from 0.01 to 1 μg/mL possessed good biocompatibility.

### Protein adsorption

Studies have indicated that protein can be absorbed onto the surface of many types of biomaterials^33–37^. Interactions between protein and biomedical materials might not only change the structure and function of proteins but also affect the protein biological activity. Therefore, it is essential to study the adsorptive properties of materials to protein. Here, the adsorption of Janus microspheres to BSA is shown in Fig. 8(c). The ratio of protein adsorbed increased gradually with an increasing Janus microsphere concentration; in particular, when the concentration reached 3.2 mg/mL, the adsorbed protein concentration was as high as 0.089 mg/mL. This finding demonstrated that the proper concentration of Janus microspheres was extremely important to use or reduce the influence of protein adsorption. Further studies will be carried out to investigate in depth the protein adsorption phenomenon following the experiments described in Salvador-Morales, and Cerdervall *et al*.^36,37^.

### Hemolytic assay

The hemolytic properties of dispersion liquids with different contents of Janus microspheres were evaluated, and the results are shown in Fig. 8(d–f). The image in Fig. 8(e) shows that the positive control was a transparent solution and that other groups were epinephelos. After 2 h centrifugation, the positive control was still transparent, and other groups demonstrated obvious red precipitation (Fig. 8(f)), which indicates that Janus microspheres caused no remarkable hemolysis. The hemolysis ratios were measured and presented in Fig. 8(d). The hemolysis ratio increased with the increase of Janus microsphere concentrations. Significant differences in the hemolysis ratio were found between concentrations 0.1/1 and concentration 100 μg/mL, which suggests the importance of appropriate microsphere concentrations. However, the hemolysis ratios of Janus microspheres with different concentrations of 0.1, 1, 10, and 100 μg/mL were only 0.62%, 0.71%, 1.1%, and 1.6%, respectively, which is much lower than the international standard of 5%^38,39^. The above results suggested that the Janus microspheres may exhibit good blood-compatibility.

## Conclusion

In this study, magnetic-fluorescent bifunctional Janus microspheres and composite microspheres were prepared using electrospraying with both double needles and single needles, in which TbLa_3_(Bim)_12_ and Fe_3_O_4_ MNPs modified by HCl were employed. Compared to Tb(Bim)_3_ with a single RE ion, TbLa_3_(Bim)_12_ exhibited a stronger fluorescent intensity due to the existence of the inert RE ion La^3+^. Janus microspheres exhibited a good Janus structure, and the drug TbLa_3_(Bim)_12_ with dual RE ions and Fe_3_O_4_ MNPs was distributed in different chambers. These Janus microspheres displayed a much higher fluorescent intensity than did the composite microspheres. Moreover, the fluorescent intensity of Janus microspheres was greatly influenced by the contents of TbLa_3_(Bim)_12_ and Fe_3_O_4_ MNPs. Therefore, it is quite important to optimize the content of TbLa_3_(Bim)_12_ and Fe_3_O_4_ MNPs to obtain DDS microspheres with good magnetic-fluorescent performance. This study also indicated that both Janus microspheres and composite microspheres exhibited good magnetic properties, thermal stability and biocompatibility. Janus microspheres [PLGA/TbLa_3_(Bim)_12_]//[PLGA/Fe_3_O_4_] with enhanced fluorescent properties present numerous potential applications for drug delivery, biological probing, and bioimaging.

## Data Availability

The authors declare that the main data supporting the findings of this study are available within this article.
